# Effect of Parboiling Conditions on Physical and Cooking Quality of Selected Rice Varieties

**DOI:** 10.1155/2020/8810553

**Published:** 2020-09-08

**Authors:** Ayenew Meresa, Ayalew Demissew, Seifu Yilma, Getu Tegegne, Kiber Temesgen

**Affiliations:** Amhara Agricultural Research Institute (ARARI), P.O. Box 527, Bahir Dar, Ethiopia

## Abstract

Most locally cultivated rice varieties in Ethiopia have low physical (low head rice yield, high broken rice yield, and high percentage of chalkiness) and cooking qualities (low water uptake ratio and swelling ratio). Parboiling, a process which involves soaking, steaming, and drying, has been identified as a key technique to improve cooking and milling quality of rice. The current study is aimed at elucidating the effect of parboiling on physical and cooking qualities of three rice varieties (Gumara, Edget, and Narica4) collected from Fogera National Rice Research and Training Center, Amhara region, Ethiopia. Each rice variety was subjected to different soaking temperatures (40°C, 50°C, 60°C, 70°C, and 80°C) and steaming time (10, 20, 30, 40, and 50 minutes). The treatment effect results indicated that parboiling has a significant effect (*P* < 0.05) on head rice yield and percentage of broken rice with increased soaking temperature and steaming time as compared to the control. For instance, percent head rice yield increased as soaking temperature (from 40 to 80°C) and steaming time (from 10 to 50 min) increased: for Gumara, from 4.07 to 93.6%, for Edget, 9.47 to 96.53, and from 3.20 to 91.67 for Narica4. Percentage chalkiness had decreased as soaking temperature and steaming time increased: 97.33% to 0.00% for Gumara, 97.80% to 0.00% for Edget, and 100.00% to 0.13% for Narica4 as compared to 100% for control of all varieties. The minimum cooking time was identified as 16-23 min for Gumara, 16-23 min for Edget, and 15-20 min for Narica4 rice varieties. The result of the present study clearly showed that parboiling with high soaking temperature and steaming time increased the head rice yield, water uptake ratio, decreased percentage chalkiness, and enhanced the overall quality of the rice varieties.

## 1. Introduction

Rice (*Oryza sativa* L.) is a sole cereal crop cooked and consumed mainly as whole grain and hence considerations on grain quality are much more relevant than other food crops [1]. Rice supplies high-value carbohydrates accounting for more than 50% of the daily calorie intake, and it is consumed by more than 67% of the world's population [2, 3].

According to the Central Statistical Agency [4] of Ethiopia report, the number of farmers engaged in rice production was about 115 thousand in 2012/13, and it has increased to about 161 thousand in 2017/18 [5]. Similarly, the area covered showed an increment from about 41 thousand ha in 2012/13 [4] to about 53 thousand ha in 2017/18 [5] along with increased production from about 121 thousand tons in 2012/13 [4] to 151 thousand tons in 2017/18 [5]. Although the production shows an increment through time, there is a substantial postharvest loss during rice milling. Furthermore, the local rice has low market price due to its poor cooking quality due to high breakage during milling, chalky grains, low head rice, and low water absorption capacity.

Parboiling is a hydrothermal process consisting of soaking, heating, and drying operations modifying the qualitative and processing behavior of rice [6, 7]. Soaking is a hydration process in which the diffusion-controlled water uptake migrates into the rice kernel [8], and continuous heating leads to nonreversible swelling and fusion of starch granules. Due to the reason that starch granules are gelatinized, followed by relevant reassociation, different changes occur in rice that plays an important role in various postharvest handling and processing operations, such as storage, milling, cooking, and eating qualities [9]. Due to the diversity in genetic and environmental factors [10, 11], different rice varieties vary in their cooking and sensory characteristics [12].

Locally milled rice in Ethiopia is poor in quality, usually consumed in rural areas, and cannot compete with imported rice both in terms of price and quality. The poor cooking quality of the local rice makes urban consumers to prefer imported rice to local ones. Hence, there is a need to improve the quality of locally produced rice in terms of physical and cooking attributes of the grain in order to make it competitive with imported ones.

## 2. Materials and Methods

### 2.1. Sampling

About 50 kilograms of paddy rice of each selected rice variety (Narica-4, Edget, and Gumara) harvested in 2017 and stored for six months was collected from Fogera National Rice Research and Training Center, where the milling activity was also performed. Experiments on parboiling processes, physical quality analysis, and the cooking quality analysis were conducted at the Food Science and Post-Harvest Handling Research Directorate laboratory, Amhara Agricultural Research Institute, Bahir Dar, Ethiopia.

### 2.2. Experimental Design

The experiment consisted of two treatments with five levels and one control. Soaking temperature (°C) and steaming time (minutes) were the treatments of the experiment. Five temperatures (40, 50, 60, 70, and 80°C) at a constant soaking time and five steaming durations (10, 20, 30, 40, and 50 min) were the levels of the treatments. A complete randomized design was employed for the levels and each was replicated three times.

### 2.3. Parboiling Process

The laboratory parboiling procedure was conducted according to the method described by Danbaba et al. [13] with some modification. A laboratory water bath (Clifton unstirred bath, England) with temperature regulation of ±2°C was used for soaking rice grains before steaming to produce parboiled rice. About 14 kg of rice sample was put in a perforated dish and soaked in a water bath. Soaking was done in hot water of five different temperatures (40, 50, 60, 70, and 80°C) at constant soaking time. Then the soaked sample was steamed for five steaming durations (10, 20, 30, 40, and 50 min) over boiling water. After steaming, the parboiled paddy was spread on a tray with a thickness of about 2 cm at ambient condition for about 4 days to dry until equilibrated to 12-14% moisture content. The dried parboiled rice sample was dehusked (TYPE 25 M, Oya Tanzo Manufacturing Co., Ltd, Japan), polished (CBS550BS, SATAKE, Japan), and packed with plastic bags till analysis.

### 2.4. Determination of Physical Characteristics

#### 2.4.1. Moisture Content

Grain moisture content was measured in triplicates using a digital moisture meter (Riceter J301, KETT, Japan).

#### 2.4.2. Husking/Milling Quality

From 1.2 kg of paddy rice, it was done by the removal or separation of husk and bran to recover the edible portion of rice. The milling quality (husking efficiency or recovery percentage) was calculated according to the international rice research institute method [14] as follows:

(1) $$\begin{array}{c} \text{Milling quality}\left(\%\right)=\frac{\text{Mass of milled rice}\left(\text{grams}\right)}{\text{Mass of original paddy sample}\left(\text{grams}\right)}*100. \end{array}$$

#### 2.4.3. Percentage of Broken Rice

Broken rice is an estimate of those kernels that are less than ¾ of their normal length after milling (dehusking). This was determined by weighing 50 g samples of polished rice and separating into broken and unbroken fractions. This was done manually with careful handpicking and repicking. Each portion was weighed and expressed as a percentage of the initial weight of rice. This was conducted by the method followed by Adu-Kwarten et al. [15].

(2) $$\begin{array}{c} \text{Broken rice}\left(\%\right)=\frac{\text{Weight of broken grains}}{\text{Weight of taken sample}}*100. \end{array}$$

#### 2.4.4. Percentage of Head Rice

From a 50 g sample of cleaned and milled rice, the head rice was manually separated and weighed. Milled rice grains with a length greater than three quarters of complete grains were classified as head rice. The head rice yield was calculated using the method followed by Fofana et al. [16] with some modifications by calculating head rice percentage instead of head rice ratio.

(3) $$\begin{array}{c} \text{Head rice yield}\left(\%\right)=\frac{\text{Weight of head rice}}{\text{Weight of milled rice sample}}*100. \end{array}$$

#### 2.4.5. Percentage Chalkiness

Percentage of rice chalkiness was calculated from three replicates of 50 g samples, according to the method outlined by WARDA [17]. The amount of chalk in the milled rice was measured using seed viewer florescent (QUG/A2-SL, UK) for transmission of light. A perfect rice grain is translucent, allowing the transmission of light, whereas opaque or chalky areas in a chalky grain prevent this transmission. Chalkiness is expressed as the proportion of opaque relative to translucent areas in rice grains.

(4) $$\begin{array}{c} \text{Chalkiness}\left(\%\right)=\frac{\text{Weight of chalky grain}}{50\text{g samples}}*100. \end{array}$$

#### 2.4.6. Determination of Cooking Properties

*(1) Minimum Cooking Time (MCT)*. This was done according to Singh et al. [11]. About 2 g head rice samples was taken in a test tube and cooked in 20 ml distilled water in a boiling water bath. The cooking time was determined by removing a few kernels at different time intervals during cooking and pressing them between two glass plates until no white core was left

*(2) Water Uptake Ratio*. Water uptake ratio of cooked rice was determined by the increase in weight of rice after subjecting it to MCT as described above. Eight grams of rice was cooked with 100 ml water in a 200 ml cylinder on an electric heater [18]. Water uptake ratio was calculated as

(5) $$\begin{array}{c} \text{Water uptake ratio}=\frac{\text{Weight of cooked rice}}{\text{Weight of raw rice}}. \end{array}$$

*(3) Swelling Ratio*. Milled rice (8 g) was placed into a wire mesh cooking basket. The height of the raw rice in the cooking basket was measured using a digital caliper (SS17DV150, China) (H1). The samples were cooked according to the cooking times determined above. The cooking basket was subsequently removed and stood erect for 2 minutes for the water to drain off. The height of the cooked rice in the cooking basket was measured using a digital caliper (H2). This determination was carried out in triplicate [16]

(6) $$\begin{array}{c} \text{Swelling ratio}=\frac{\text{Height of cooked rice}\left(\text{H}2\right)}{\text{Height of raw rice}\left(\text{H}1\right)}. \end{array}$$

#### 2.4.7. Data Analysis

Data were analyzed using the SAS software version 9.0 and one-way analysis of variance (ANOVA) followed by Duncan's multiple range test for the multiple comparison analysis was carried out. Statistical significant test was carried out at 0.05 probability level.

## 3. Result and Discussion

### 3.1. Physical Characteristics of Rice Varieties

The mean moisture content for paddy Gumara rice ranged from 12.47% to 15.13%, from 14% to 15% for Edget, and for Narica4, it ranged from 12.10% to 14.73% (Table 1). There was significant difference between the combination of each soaking temperature and steaming time treatments (*P* < 0.05) for the three varieties (Table 1). In general, the moisture contents of the tested rice varieties were comparable with those reported by Bleoussi et al. [19] (14%), Farhan et al. [20] (10-12%), Adu-Kwarten et al. [15] (14%), Prasad et al. [21] (13-14%), and Ayamdoo et al. [22] (15%).

Table 1 shows that there was significant difference (*P* < 0.05) within each soaking temperature and steaming time treatment combination for the three rice varieties with respect to head rice yield. In general, the head rice yield of the three rice varieties had increased as the soaking temperature and steaming time increased. Gumara, Edget, and Narica4 rice varieties soaked at 80°C and steamed for 50 minutes gave the highest yield and best quality rice with a mean value of 93.60%, 97.93%, and 91.67%, respectively. According to Musa et al. [23], head rice yield is the current standard to assess commercial rice milling quality, and hydrothermal treatment increases head rice yield which then increases the quality indexes of processed rice [24].

The mean value of broken grains decreased as the soaking temperature and steaming time increased for all the three rice varieties. For instance, at 80°C and 50 min treatment, the percentage of broken rice was 6.4% (68.53% for control), 2.07% (70.47% for control), and 8.33% (90.3% for control) for Gumara, Edget, and Narica4 rice varieties, respectively. Different researchers also reported the good effect of parboiling in reducing the percentage of broken rice during milling. For instance, Ayamdoo et al. [22] reported that broken grains significantly decrease from 47% of control samples to 9.5% of parboiled rice. Prasad et al. [21] also reported that percentage of broken rice was significantly reduced from 27.25% for control to 6.31% for parboiled treatment.

The mean percentage value of chalkiness for Gumara rice variety ranged from 0 to 97.33%; for Edget, it ranged from 0 to 97.80% and Narica4 from 0.13 to 100% as compared to almost 100% for the control of all rice varieties. There was a significant difference (*P* < 0.05) with respect to chalkiness in all rice varieties within the treatment combinations. Less mean percentage chalkiness value for Gumara rice variety (1.53%-0.0%) between 70°C, 10' and 80°C, 50'; Edget variety (0.87%-0.0%) between 60°C, 50' and 80°C, 50'; and Narica4 rice variety (0.93%-0.13%) between 70°C, 50' and 80°C, 50' was recorded. Generally, the result this study shows that as the soaking temperature and steaming time increased, the percentage chalkiness decreased. As a result, the endosperm translucency, which is an acceptable quality parameter for rice, was enhanced. The translucency character of the endoderm increases during parboiling treatment, mainly due to the pregelatinization of its starch [25]. This character of the endosperm mostly determines the appearance of the grain, and this is inversely related to the amount of chalkiness. Parboiling and cooking processes disappear partially or totally the chalkiness of rice, which may have no direct effect on cooking and eating qualities. But a large amount of chalkiness downgrades the physical quality, reduces milling recovery, and can determine attractiveness on a competitive price on the market [15, 26–28].

As indicated in Table 2, Edget rice had the highest percentage head rice (60.4%) compared to the other two rice varieties. Similarly, less percentage of broken rice (39.6%) and chalkiness (55.18%) was recorded for Edget rice. The result of varietal effect indicated that there was no significant difference between Gumara (34.41) and Narica4 (32.84) (*P* > 0.05) rice varieties in terms of head rice yield and percentage of broken rice.

As indicated in Table 3, soaking temperature significantly affected (*P* < 0.05) the percentage of head rice, broken rice, and percentage chalkiness of the rice. As soaking temperature increased, percentage of head rice increased and percentage of broken rice reduced. Chalkiness at 40°C, 50°C, and 60°C was not significantly different from the control (*P* > 0.05) but significant differences were observed at higher temperatures (70°C and 80°C). Similarly, as steaming time increased, percentage of head rice increased and percentage of broken rice reduced. In addition, steaming time significantly affected (*P* < 0.05) the percentage chalkiness of rice.

### 3.2. Cooking Properties of Rice Varieties

The minimum cooking time for Gumara ranged from 16 min (at 40°C, 10') to 23 min (at 80°C, 50'), for Edget 16 min (at 40°C, 10') to 23 min (at 80°C, 50'), and for Narica4 15 min (at 40°C, 10') to 20 min (at 80°C, 50') (Table 4). The trend revealed that when soaking temperature and steaming time increased, the cooking time also increased. The results obtained were similar with that reported by Farhan et al. [20] (20.67 min for nonparboiled rice to 25.00 for parboiled rice), Tetens et al. [29] (15.42-17.20 min for raw and for parboiled rice 20.70-23.05 min), Otegbayo et al. [30] (56 min for parboiled and 49 min for nonparboiled rice), and Kar et al. [31] (22 min for parboiled and 15 min for raw rice). Cooking time of parboiled rice was longer than the nonparboiled rice because of the strong cohesion between endosperm cells that makes the tightly packed starch granules to hydrate at a slower rate, which leads to a decreased in-water penetration into the grain [30].

As shown in Table 4, the result of water uptake ratio for Gumara rice variety shows that there was no significant difference among each soaking temperature and steaming time treatment combination (*P* > 0.05) from (40°C, 20') to (80°C, 50'). But the mean value of water uptake ratio within the treatment combination is ranged from 3.33 at (40°C, 20') to 3.47 at (80°C, 50') with the control value of 3.23. There was no significant difference throughout the treatment combination, including the control for Edget variety (*P* > 0.05). The mean value within the treatment combination is ranged from 3.12 at (40°C, 10') to 3.40 at (80°C, 50') with the control value of 3.11. There was a significant difference between the treatment combinations of Narica4 rice variety (*P* < 0.05). The mean value within the treatment combination is ranged from 3.45 at (80°C, 30') to 5.08 at (50°C, 50') with the control value of 4.45. Among those three varieties, Narica4 rice variety achieved the highest mean value of the water uptake ratio. In this study, for Narica4 rice variety, no clear trend was observed with the changes in the water uptake ratio at different storage of treatment combination; however, the results clearly showed that parboiling drastically increased the water uptake ratio of the rice. Hence, for Gumara and Edget varieties, increasing the soaking temperature and soaking time increases the mean value of water uptake ratio. Similar to the result obtained by Otegbayo et al. [30], the water absorption of the parboiled rice was higher (13.56 ml/g) than that of the nonparboiled rice (10.31 ml/g). Kurien et al. *[*31*]* reported that water absorption capacity, as reflected by the swelling ratio, is significantly low for parboiled rice as compared with raw rice cooked for the same period (at 10 min, raw (2.22) to parboiled (2.06)). However, the samples of raw and parboiled rice cooked to an equivalent degree of softness show that parboiled rice can absorb more water without losing its shape (from 2.06 at 10 min to 3.55 at 40 min) [32]. Mustapha [33] indicates that parboiled rice has higher water absorption, which may be a result of the steaming pressure during parboiling, which in turn, affects starch gelatinization.

The mean value of swelling ratio for Gumara rice variety was ranged from 2.46 at 40°C, 10' to 3.00 at 80°C, 50' with the control mean value of 2.77 (Table 4). The data revealed that there was a significant difference (*P* < 0.05) within the treatment combination, including the control. The trend of this data for specific Gumara rice variety indicates that the swelling ratio had increased as the soaking and steaming time were increasing. Also, the mean value of Edget rice variety was ranged from 1.11 to 3.15, and that indicates there was a significant difference (*P* < 0.05). There was no clear trend observed for the swelling ratio data of Edget variety. Narica4 rice variety has mean value ranged from 2.94 at 60°C, 40' to 5.22 at 40°C, 50' with the control mean value of 4.71. In this study, for Narica4 rice variety, no clear trend was observed with the changes in the swelling ratio at different storage of treatment combination; however, the results clearly showed that parboiling drastically increases the swelling power of the parboiled rice. Kurien et al. [32] reported that the swelling ratio is significantly low for parboiled rice as compared with raw rice cooked for the same period (at 10 min, raw (2.57) to parboiled (2.32)). However, the samples of raw and parboiled rice cooked to an equivalent degree of softness show that parboiled rice can absorb more water without losing its shape (from 2.32 at 10 min to 4.54 at 40 min) [32].

There was a significant difference (*P* < 0.05) in minimum cooking time, water uptake ratio, and swelling ratio among the three varieties (Table 5). The minimum cooking time was recorded for Gumara (19 : 00 min), whereas the highest water uptake and swelling ratio were recorded for Narica4 variety which were 4.20 and 4.28, respectively (Table 5). As indicated in Table 6, some soaking temperatures had significantly affected (*P* < 0.05) the water uptake ratio and swelling ratio of the tested rice. But all steaming times, including the control, did not significantly affected (*P* > 0.05) the water uptake ratio and swelling ratio.

## 4. Conclusion and Recommendation

The study explored the effect of parboiling on physical and cooking qualities of three rice varieties, Gumara, Edget, and Narica4. Higher soaking temperatures and steaming times increased the head rice yield, water uptake ratio, and swelling ratio and decreased chalkiness and broken rice which are indicators of good quality rice. On the other hand, the swelling ratio for Edget rice and water uptake ratio and swelling ratio for Narica4 variety had no clear trend at treatment conditions. Higher treatment conditions increased the cooking time of parboiled rice varieties compared to the cooking time of nonparboiled rice. In general, parboiling with prolonged soaking temperature and steaming time improved the physical and cooking quality of local rice varieties.

## Figures and Tables

**Table 1:** Physical property of rice varieties at different soaking temperatures and steaming time.

Soaking T (°c)	Steaming time(min)	Gumara rice					Edget rice					Narica4 rice				
		Husking quality %	Moisture content %	% head rice	% broken	% chalkiness	Husking quality %	Moisture content %	% head rice	% broken	% chalkiness	Husking quality %	Moisture content %	% head rice	% broken	% chalkiness
40	10	78.1	15.13^a^	4.07*^Θ^*′	95.93^!^	97.33*^β^*	79.30	14.57^bcd^	13.13^Ϙ^′*^Φ^*′	86.87^±!^	97.80*^β^*	80.00	13.37^dfe^	3.20*^Θ^*	96.80^!^	100.00*^α^*
40	20	82.7	14.70^bdc^	8.80^9^	91.20^±^	96.53*^γβ^*	79.50	14.27^cde^	11.60^Ϙ^′*^Φ^*′	88.40^±!^	96.80*^βγ^*	80.20	13.03^gf^	5.07^9*Θ*^′	94.93^±!^	99.67*^αβγ^*
40	30	77.8	13.23^ih^	10.93^98^	89.07^#±^	96.47*^γβ^*	80.00	14.17^de^	9.47^Ϙ^′	90.53^!^	96.00*^γδ^*	80.20	12.33^h^	6.20^9*Θ*^′	93.80^±!^	99.60*^αβγ^*
40	40	78.1	14.67^dc^	12.67^7^′^98^	87.33^#ͷ ± $^	96.33*^γβ^*	79.90	14.70^abc^	19.00*Δ*′	81.00^#^	96.07*^γδ^*	80.00	12.10^h^	8.07^98^	91.93^±#^	99.33*^αβγ^*
40	50	77.8	14.50^efd^	13.33^7958^	86.67^#ͷ ± $^	96.07*^γβδ^*	79.60	14.47^bcd^	67.47^67^	32.53^∗^^ͻ^	97.00*^βγ^*	80.20	13.43^dfe^	14.67^7^	85.33^$^	99.33*^αβγ^*
50	10	78.6	13.23^ih^	9.87^98^	90.13^#±^	96.00*^γβδ^*	79.70	14.23^de^	12.13^Ϙ^′*^Φ^*′	87.87^±!^	96.53*^βγ^*	80.10	14.33^ba^	5.13^9*Θ*^′	94.87^±!^	99.60*^αβγ^*
50	20	78.3	14.97^bac^	14.07^768^	85.93^^#ͷ$^	95.60*^γεβδ^*	79.70	14.53^bcd^	14.47*^Φ^*′	85.53^±^	95.60*^γδε^*	80.60	14.57^a^	6.00^9*Θ*^′	94.00^±!^	99.47*^αβγ^*
50	30	78.6	14.50^efd^	15.93^768^	84.07^^ͷ$^	95.47*^γεβδ^*	79.50	14.43^bcd^	51.07^8^	48.93^	94.53*^ε^*	80.20	14.73^a^	19.67^6^	80.33*^ε^*	99.40*^αβγ^*
50	40	78.5	14.57^efdc^	17.00^76^	83.00^^ͷ^	94.47*^γεδ^*	79.90	14.33^bcde^	69.00^65^	31.00^∗^^ͻ<^	94.40*^ε^*	76.60	13.67^dce^	20.00^6^	80.00*^ε^*	99.67*^αβγ^*
50	50	79.0	14.33^efd^	17.73^6^	82.27^	93.87*^εζδ^*	80.10	14.30^bcde^	70.20^65^	29.80^∗^^<^	95.07*^εδ^*	81.20	14.63^a^	25.20^5^	74.80^	99.40*^αβγ^*
60	10	78.3	14.60^efdc^	11.33^98^	88.67^# ± $^	93.53*^εζ^*	79.70	14.40^bcde^	28.80*^Θ^*′	71.20^$^	94.33*^ε^*	80.00	14.67^a^	6.87^9^	93.13^±^	99.53*^αβγ^*
60	20	78.5	14.33^efd^	14.80^768^	85.20^^#ͷ$^	92.20*^ζ^*	78.70	14.23^de^	45.53^9^	54.47^ͷ^	94.20*^ε^*	76.10	13.83^dc^	13.80^7^	86.20^$^	99.13*^βγ^*
60	30	78.5	14.47^efd^	17.53^76^	82.47^^ͷ^	92.00*^ζ^*	78.00	14.43^bcd^	71.40^5^	28.60^<^	92.20*^ζ^*	80.20	13.50^dfe^	23.73^5^	76.27^	99.13*^βγ^*
60	40	75.7	12.47^k^	26.33^5^	73.67^ͻ^	92.07*^ζ^*	78.00	14.33^bcde^	72.27^5^	27.73^<^	92.00*^ζ^*	80.10	13.03^gf^	32.00^4^	68.00^ͻ^	99.67*^αβγ^*
60	50	78.7	14.50^efd^	42.80^3^	57.20^<^	89.13*^η^*	80.00	15.00^a^	88.93^4^	11.07^>^	0.60*^η^*	80.20	13.83^dc^	33.07^4^	66.93^ͻ^	99.13*^βγ^*
70	10	78.7	14.23^ef^	27.73^54^	72.27^∗^^ͻ^	1.53*^θ^*	74.50	14.73^ab^	65.93^7^	34.07^ͻ^	0.87*^η^*	80.10	13.47^dfe^	13.27^7^	86.73^$^	99.80*^αβ^*
70	20	77.8	12.77^kj^	46.87^3^	53.13^<^	0.00*^θ^*	79.90	14.23^de^	71.40^5^	28.60^<^	0.33*^η^*	79.90	14.10^bc^	25.80^5^	74.20^	99.73*^αβγ^*
70	30	78.7	14.20^f^	55.80^2^	44.20^>^	0.00*^θ^*	79.80	14.73^ab^	89.87^43^	10.13^+>^	0.27*^η^*	78.70	13.67^dce^	50.87^3^	49.13^∗^	99.73*^αβγ^*
70	40	73.5	12.90^ij^	57.40^2^	42.60^>^	0.00*^θ^*	79.70	14.50^bcd^	92.07^432^	7.93^+ > <^	0.13*^η^*	89.60	13.27^gfe^	49.40^3^	50.60^∗^	98.93*^γ^*
70	50	78.4	14.63^edc^	60.00^2^	40.00^>^	0.00*^θ^*	79.90	14.53^bcd^	93.33^132^	6.67^+¢-^	0.00*^η^*	79.60	13.43^dfe^	61.40^2^	38.60^<^	0.93*^δ^*
80	10	78.6	15.07^ba^	42.80^3^	57.20^<^	0.00*^θ^*	79.30	14.47^bcd^	95.07^102^	4.93^=¢-^	0.00*^η^*	78.90	13.23^gfe^	81.47^1^	18.53^>^	0.60*^δε^*
80	20	78.4	13.13^ihj^	74.40^3^	25.60^+^	0.00*^θ^*	79.70	14.50^bcd^	96.53^10^	3.47^=¢^	0.00*^η^*	79.60	13.70^dce^	82.67^1^	17.33^>^	0.67*^δε^*
80	30	78.5	13.37^h^	75.40^1^	24.60^+^	0.00*^θ^*	80.20	14.53^bcd^	96.93^10^	3.07^=¢^	0.00*^η^*	80.10	14.03^bc^	81.67^1^	18.33^>^	0.80*^δε^*
80	40	78.5	12.90^ij^	91.87^0^	8.13^−^	0.00*^θ^*	79.90	14.43^bcd^	97.20^0^	2.80^=^	0.00*^η^*	78.80	13.53^de^	83.13^1^	16.87^>^	0.27*^δε^*
80	50	78.4	13.23^ih^	93.60^0^	6.40^−^	0.00*^θ^*	79.40	14.00^e^	97.93^0^	2.07^=^	0.00*^η^*	79.20	13.53^de^	91.67^0^	8.33^+^	0.13*^ε^*
Control		78.4	13.73^g^	31.47^4^	68.53^∗^	100.00*^α^*	80.10	12.83^f^	29.53*^Θ^*′	70.47^$^	99.87*^α^*	80.10	12.87^g^	9.87^8^	90.13^#^	100.00*^α^*
	CV	-	1.56	7.70	4.04	2.10	-	1.53	3.50	5.33	1.41	-	1.89	5.24	2.56	0.53
	*F* value		40.09	313.59	313.59	4401.95		8.80	716.53	716.53	11451.8		32.48	889.31	889.31	33357.00

^∗^Means with the same alphabet, number, symbol, and Greek letter as superscript within same columns are not significantly different at 5% significance level.

**Table 2:** Overall effects of varieties on physical properties.

Variety	Husking quality %	Moisture content	% head rice	% broken	% chalkiness
Edget	78.62^ba^	14.38^0^	60.40^!^	39.60*^β^*	55.18^b^
Gumara	78.28^b^	14.01^1^	34.41^±^	65.59*^α^*	58.41^b^
Narica4	80.02^a^	13.62^2^	32.84^±^	67.16*^α^*	76.68^a^
CV	3.66	4.24	25.41	18.82	30.87
*F* value	2.65	31.63	159.84	159.84	27.34

^∗^Means with the same alphabet, number, symbol, and Greek letter as superscript within columns are not significantly different at 5%.

**Table 3:** Effect of soaking and steaming time on the physical properties of rice varieties (Gumara, Edget, and Narica4).

		Moisture content	% head rice	% broken	% chalkiness
Soaking T°(°C)	40	13.91^b^	13.84^4^	86.16^!^	97.62*^α^*
	50	14.38^a^	24.50^3^	75.50^±^	96.60*^α^*
	60	14.11^ba^	35.28^2^	64.72^#^	88.59*^α^*
	70	13.96^b^	57.41^1^	42.59^$^	26.82*^β^*
	80	13.84^b^	85.49^0^	14.51^ͷ^	0.16*^γ^*
	Control	13.14^c^	23.62^3^	76.38^±^	100*^α^*
	CV	4.24	25.41	18.82	30.87
	*F* value	5.79	314.20	314.20	241.63

Steaming time (min)	10	14.25^a^	28.05^3^	71.95^!^	65.16*^β^*
	20	14.06^a^	35.45^2^	64.55^±^	64.66*^β^*
	30	14.02^a^	45.10^1^	54.90^#^	64.37*^β^*
	40	13.69^b^	49.83^1^	50.17^#^	64.22*^β^*
	50	14.18^a^	58.09^0^	41.91^$^	51.38*^γ^*
	Control	13.14^c^	23.62^3^	76.38^!^	100*^α^*
	CV	4.24	25.41	18.82	30.87
	*F* value	5.87	53.76	53.76	4.12

^∗^Means with the same alphabet, number, symbol, and Greek letter as superscript within columns are not significantly different at 5% significance level.

**Table 4:** Cooking property of rice varieties at different soaking temperature and steaming time.

Soaking T (°c)	Steaming time (min)	Gumara			Edget			Narica4		
		Minimum cooking time	Water uptake ratio	Swelling ratio	Minimum cooking time	Water uptake ratio	Swelling ratio	Minimum cooking time	Water uptake ratio	Swelling ratio
40	10	16	3.27^bc^	2.46*^θ^*	16	3.12^a^	2.11*^ηθ^*	15	3.70^igh^	4.59*^ε^*
40	20	16	3.33^bac^	2.46*^θ^*	16	3.25^a^	1.88*^ι^*	15	4.31^edf^	3.02*^ι^*
40	30	16	3.34^bac^	2.47*^ηθ^*	16	3.23^a^	2.29*^η^*	16	3.61^ih^	3.22*^ιθ^*
40	40	17	3.34^bac^	2.53*^ηζθ^*	16	3.15^a^	2.33*^ηζ^*	16	3.67^igh^	5.08*^βαγ^*
40	50	17	3.35^bac^	2.55*^ηζθ^*	18	3.15^a^	2.10*^ηθ^*	16	3.93^ghf^	5.22*^α^*
50	10	17	3.26^bac^	2.57*^ηζθ^*	18	3.18^a^	2.20*^η^*	16	3.98^eghf^	3.25*^ιθ^*
50	20	17	3.33^bac^	2.58*^ηεζθ^*	18	3.16^a^	2.14*^η^*	17	4.77^bac^	4.88*^εβδαγ^*
50	30	17	3.35^bac^	2.58*^ηεζθ^*	18	3.18^a^	1.11*^λ^*	17	4.08^egdf^	5.16*^βαγ^*
50	40	17	3.36^bac^	2.59*^ηεζθ^*	18	3.18^a^	1.50*^κ^*	17	4.75^bac^	4.09*^ζ^*
50	50	18	3.37^bac^	2.60*^ηεζθ^*	18	3.21^a^	1.73*^ι^*	17	5.08^a^	4.76*^εβδγ^*
60	10	19	3.36^bac^	2.61*^ηεζθ^*	19	3.20^a^	1.82*^ι^*	17	4.96^a^	4.58*^ε^*
60	20	19	3.38^bac^	2.58*^ηεζθ^*	19	3.21^a^	2.65*^εδ^*	17	4.07^egdf^	5.17*^αγ^*
60	30	20	3.38^bac^	2.60*^ηεζθ^*	19	3.15^a^	2.76*^εδγ^*	17	5.03^a^	4.92*^εβδαγ^*
60	40	20	3.39^bac^	2.60*^ηεζθ^*	19	3.24^a^	2.60*^ε^*	17	3.68^igh^	2.94*^ι^*
60	50	20	3.38^bac^	2.61*^ηεζθ^*	19	3.23^a^	2.86*^βδγ^*	17	4.78^bac^	4.88*^εβδαγ^*
70	10	20	3.42^bac^	2.63*^εζδ^*	21	3.36^a^	3.04*^βα^*	18	3.97^eghf^	3.47*^ηθ^*
70	20	20	3.42^bac^	2.62*^ηεζδ^*	21	3.30^a^	3.15*^α^*	18	4.82^ba^	3.83*^ηζ^*
70	30	20	3.42^bac^	2.64*^εζδ^*	22	3.33^a^	2.90*^βγ^*	19	4.41^dc^	3.36*^ιθ^*
70	40	21	3.43^ba^	2.64*^εζδ^*	22	3.35^a^	2.53*^εζ^*	19	4.35^ed^	3.14*^ιθ^*
70	50	21	3.44^ba^	2.67*^γεζδ^*	22	3.35^a^	1.41*^κ^*	19	3.95^eghf^	4.62*^εδ^*
80	10	21	3.45^a^	2.67*^γεζδ^*	22	3.36^a^	2.75*^εδγ^*	19	4.35^ed^	4.61*^ε^*
80	20	22	3.46^a^	2.73*^γεβδ^*	23	3.33^a^	2.31*^ηζ^*	20	3.64^ih^	4.08*^ζ^*
80	30	22	3.46^a^	2.81*^γβ^*	23	3.37^a^	2.66*^εδ^*	20	3.45^i^	4.13*^ζ^*
80	40	22	3.46^a^	2.86*^β^*	23	3.38^a^	3.07*^βα^*	20	3.57^ih^	5.06*^βδαγ^*
80	50	23	3.47^a^	3.00*^α^*	23	3.40^a^	2.98*^βαγ^*	20	3.70^igh^	4.63*^εδ^*
Control		16	3.23^c^	2.77*^γβδ^*	16	3.11^a^	2.09*^ηθ^*	15	4.45^bdc^	4.71*^εδγ^*
	CV	-	3.07	3.15	-	7.53	5.71	-	5.13	5.46
	*F* value		1.24	6.45		0.42	50.21		16.30	31.73

^∗^Means with the same alphabet and Greek letter as superscript within columns are not significantly different at 5%.

**Table 5:** Overall effects of varieties on cooking properties of rice varieties.

Variety	Minimum cooking time (min)	Water uptake ratio	Swelling ratio
Edget	19.42^a^	3.25^2^	2.35^#^
Gumara	19.00^b^	3.38^1^	2.63^±^
Narica4	17.46^c^	4.20^0^	4.28^!^
CV	3.26	8.96	17.25
*F* value	74.93	199.19	301.48

^∗^Means with the same alphabet, number, and symbol as superscript within columns are not significantly different at 5%.

**Table 6:** Effect of soaking temperature and steaming time on cooking properties for Gumara, Edget, and Narica4 rice varieties.

		Water uptake ratio	Swelling ratio
Soaking T°(°C)	40	3.45^b^	2.95^1^
	50	3.68^a^	2.92^1^
	60	3.71^a^	3.21^10^
	70	3.69^a^	2.98^1^
	80	3.52^ba^	3.36^0^
	Control	3.59^ba^	3.19^10^
	CV	8.96	17.25
	*F* value	5.82	5.84

Steaming time (min)	10	3.59^a^	3.02^0^
	20	3.65^a^	3.07^0^
	30	3.59^a^	3.04^0^
	40	3.57^a^	3.04^0^
	50	3.65^a^	3.24^0^
	Control	3.59^a^	3.19^0^
	CV	8.96	17.25
	*F* value	0.71	1.27

^∗^Means with the same alphabet as superscript and number within columns are not significantly different at 5% significance level.

## Data Availability

The husking quality (%), moisture content (%), head rice yield (%), broken rice (%), chalkiness (%), minimum cooking time (min), water uptake ratio, and swelling ratio data used to support the findings of this study are included within the article.
